# Identifying anti-growth factors for human cancer cell lines through genome-scale metabolic modeling

**DOI:** 10.1038/srep08183

**Published:** 2015-02-02

**Authors:** Pouyan Ghaffari, Adil Mardinoglu, Anna Asplund, Saeed Shoaie, Caroline Kampf, Mathias Uhlen, Jens Nielsen

**Affiliations:** 1 Department of Biology and Biological Engineering, Chalmers University of Technology, SE-412 96, Gothenburg, Sweden; 2 Department of Immunology, Genetics and Pathology, Science for Life Laboratory, Uppsala University, SE-751 85, Uppsala, Sweden; 3 Science for Life Laboratory, KTH - Royal Institute of Technology, SE-171 21, Stockholm, Sweden; 4 Department of Proteomics, KTH - Royal Institute of Technology, SE-106 91, Stockholm, Sweden

## Abstract

Human cancer cell lines are used as important model systems to study molecular mechanisms associated with tumor growth, hereunder how genomic and biological heterogeneity found in primary tumors affect cellular phenotypes. We reconstructed Genome scale metabolic models (GEMs) for eleven cell lines based on RNA-Seq data and validated the functionality of these models with data from metabolite profiling. We used cell line-specific GEMs to analyze the differences in the metabolism of cancer cell lines, and to explore the heterogeneous expression of the metabolic subsystems. Furthermore, we predicted 85 antimetabolites that can inhibit growth of, or even kill, any of the cell lines, while at the same time not being toxic for 83 different healthy human cell types. 60 of these antimetabolites were found to inhibit growth in all cell lines. Finally, we experimentally validated one of the predicted antimetabolites using two cell lines with different phenotypic origins, and found that it is effective in inhibiting the growth of these cell lines. Using immunohistochemistry, we also showed high or moderate expression levels of proteins targeted by the validated antimetabolite. Identified anti-growth factors for inhibition of cell growth may provide leads for the development of efficient cancer treatment strategies.

Human cancer cell lines are widely used model systems for studying cellular mechanisms underlying cancer by analyzing their perturbation-response patterns in simplified experimental conditions^1^. Cell lines are derived from human tumors of diverse tissue origin, and have adapted to growth *in vitro* for prolonged periods. Comparative analysis of cell lines in combination with system-wide profiling techniques may disclose cross cell-type commonalities and variations of biological processes. This knowledge can be used for understanding cancer metabolism, identifying anticancer drugs, *in vitro* evaluation of predicted drug targets and outlining mechanisms of action of therapeutic agents^2^,^3^.

Advances in omics technology have enabled simultaneous measurement of molecular components interacting within complex interconnected networks and hereby provided insights into cellular functions and phenotypic states of the cells. However, analyzing genome-wide data and in particular gaining new biological knowledge, is a nontrivial effort^4^. Reconstruction of genome scale metabolic models (GEMs) can assist in this by integrating omics data in multiple layers to understand the effects of local interactions in the context of the whole network^5^,^6^,^7^. This makes GEMs a potential tool for analyzing omics data in health and disease states, and for identifying the underlying cellular mechanisms in the occurrence of complex diseases. To date, several generic human GEMs have been generated^8^,^9^,^10^,^11^ and these models have been employed for reconstruction of context-specific GEMs for healthy and cancerous cell-types. GEMs have been developed for studying the metabolic alterations between healthy and cancerous tissues^12^,^13^ as well as within cancerous tissues^14^. Moreover, personalized cancer GEMs has been reconstructed and used to capture common and specific metabolic shifts across the cancer patients and to identify selective anticancer drugs^15^.

Preserved proliferative signaling and avoidance of growth suppressors have been identified as hallmarks of cancer^16^. Adapting the cellular metabolism (e.g. increased synthesis of nucleotides, lipids and proteins) to accommodate tumor expansion is a critical feature of cancer, and has been leveraged for development of novel anticancer drugs^17^,^18^. In the present study, we used the concept of antimetabolites, which are structural analogues of endogenous metabolites. Antimetabolites subvert cellular processes by serving as inhibitors of all enzymes involved in metabolizing the associated endogeneous metabolite, and hereby dramatically affecting metabolic functions^19^,^20^. An important characteristic of antimetabolites is their potential to simultaneously inhibit multiple enzymes, and thereby reduce the growth of proliferating cells more effectively^21^. Antimetabolites are among the most common anticancer drugs since the discovery of aminopterin, an effective drug in remission of leukemia. Examples of antimetabolites are antifolates (e.g. Methotrexate), antipyrimidines (e.g. Cytarabine, 5-Fluorouracil) and antipurines (e.g. 6-Mercaptopurine).

In this study, we first followed a systematic approach and analyzed the global mRNA expression pattern of the 20,314 protein coding genes in eleven human cancer cell lines^22^. Secondly, we reconstructed functional cell line-specific GEMs (CL-GEMs) for these eleven cell lines using mRNA expression levels (RNA-Seq) together with our tINIT (task-driven Integrative Network Inference for Tissue) algorithm^15^ and Human Metabolic Reaction database (HMR)2^11^ (Figure 1A). We included known metabolic functions of the cell lines during the reconstruction process, and validated functionality of the models based on metabolite consumption and release (CORE) profiles of the cancer cell lines^1^. Thirdly, we compared CL-GEMs by using a statistical multi-comparison method as well as here defined heterogeneity scale. We also compared metabolic subsystems in the CL-GEMs based on the distribution of mRNA expression patterns of associated genes across the cell lines. Fourthly, we screened all metabolites in the HMR2 to identify essential metabolites for the growth of cell lines and predicted potential antimetabolites that can inhibit or kill their growth. We defined essential metabolites as metabolites for which their analogues (antimetabolites) may disrupt the metabolic network and lead to cell death. Each antimetabolite was also evaluated for its *in silico* toxicity by employing GEMs for 83 human healthy cell-types. Next, we experimentally tested the effect of an L-carnitine analogue, one of the potential antimetabolite that was predicted to be effective on all cell lines. Significant difference in proliferation of cultured cell lines in presence or absence of selected analogue, confirmed our computational prediction. Finally, we presented high or moderate protein staining level of the CPT1A and CPT1B, which were targeted by the L-carnitine analogue in cell lines using the antibodies generated in the Human Protein Atlas (HPA).

## Results

### Transcriptomics data of Human Cancer Cell lines

**Figure 1:**
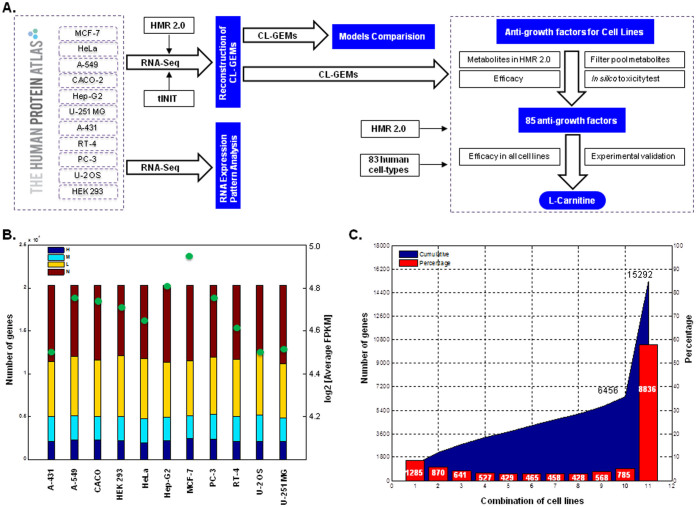
Pipeline for prediction of anti-growth factors using CL-GEMs. (A) CL-GEMs were reconstructed by introducing RNA expression profile of 20,314 protein coding genes across eleven human cancer cell lines into the HMR2 using the tINIT algorithm. CL-GEMs were used to evaluate all metabolites present in HMR2 and to identify potential anti-growth factors, which were followed by filtering the pool metabolites and analyzing *in silico* toxicity of predicted targets. Finally, one of the potential anti-growth factors were validated experimentally. (B) Protein coding genes for each cell line were categorized based on the transcript expression levels. Stacked bars represent different expression levels and filled circles shows the average FPKM value in log2 scale. (C) Distribution of 15,292 detected genes across different combination of cell lines are shown. Area curve represents cumulative number of genes through different combinations of cell lines, while bar plots shows the percentage of each combination together with corresponding number of genes inside the bar. 8,836 (~58%) of all detected genes were shared by all cell lines and remaining 42% distributed between different combinations, from which cell specific genes outnumbers the other combinations with 1,285 (~8%) of assigned genes.

RNA-Seq have been employed to identify the tissue-specific expression of genes across the major human tissues and combined with the antibody based profiling of the same tissues^23^,^24^,^25^,^26^,^27^. Previously, we also performed a comparative transcriptomics analysis based on the quantitative RNA profiling of protein-coding genes across eleven human cancer cell lines derived from distinct cellular phenotypes^22^. Fragment per kilobase of exon model per million mapped read (FPKM) values were calculated for each gene, and expression levels were categorized into high (FPKM > 50), medium (FPKM > 20) and low (FPKM > 1), and FPKM < 1 was used to define genes as *not detected*. Based on this cut-off the number of protein coding genes expressed ranged from 11,000 to 12,500 across the eleven cell lines. Approximately 43% of the expressed genes for each cell line were found in the category of high and medium RNA expression levels and the average transcript abundance score in a log2 scale ranged from 4.90 for the epidermoid carcinoma cell line (A-431) to 5.30 for the metastatic breast adenocarcinoma cell line (MCF-7) (Figure 1B). The transcript profiling of cell lines was used to study the cross-cell line distribution of detected protein coding genes (Figure 1C). In total, 15,292 out of 20,314 protein coding genes were expressed in at least one of the cell lines, from which nearly 58% (n = 8836) were commonly detectable transcripts shared by all cell lines and the remaining 42% (n = 6456) distributed in different combination of cell lines, with the highest contribution of cell specific genes (n = 1285). Through in-depth analysis of the data, we found that there is a connection between the mRNA expression levels of detected genes and their presence across cell lines. We found that genes detected in all eleven cell lines had higher transcript expression level compared to cell specific genes, with an average of 9 fold change (Supplementary Dataset 1).

### Reconstruction of GEMs for human cancer cell lines

A generic genome-scale metabolic model of cancer has been developed to capture the common metabolic functions between cancer types^28^. A core of 197 highly expressed metabolic genes common to more than 90% of the cell lines in the NCI-60 cell lines database have been employed to reconstruct a generic cancer metabolic network, and the resulting model was used to predict anti-cancer drug targets^28^. Here, we reconstructed CL-GEMs for eleven human cancer cell lines based on cell line specific RNA-Seq data, HMR2 and our task-driven reconstruction approach. Lists of 56 metabolic tasks that are known to occur in all cell types were used during the reconstruction process. These metabolic tasks can be categorized as processes for provision of energy and redox, biosynthesis of metabolites, substrate utilization and internal conversion processes (Supplementary Dataset 2). Growth was also added as a metabolic task to ensure that the cells can proliferate. The CL-GEMs were reconstructed such that the models should be able to perform all the defined tasks and at the same time maintain the consistency with the transcriptomics data. The eleven CL-GEMs are presented in the Human Metabolic Atlas portal (http://www.metabolicatlas.org/) in the Systems Biology Mark-up Language format.

**Figure 2:**
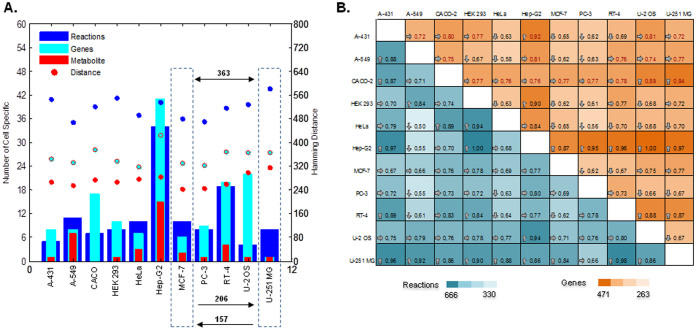
Differences between the CL-GEMs. (A) Distribution of cell specific genes, metabolites and reactions across CL-GEMs (bar plots). Filled circles represent average Hamming distance of each CL-GEM compared to other models. Here, Hamming distance is an indicator of required change to transform one model to the other based on each parameter (Supplementary Dataset 4). For example, 363 changes in genes profiles are required for inter-transformation of metastatic breast adenocarcinoma cell line (MCF-7) and glioblastoma cell line (U-251 MG), from which 206 changes in genes corresponds to transformation of MCF-7 to U-251 MG and 157 changes in genes corresponds to transformation of U-251 MG to MCF-7. (B) Pair wise comparison of CL-GEMs based on genes and reactions. Numbers inside the colored squares represent the ratio of pair wise difference between CL-GEMS compared to maximum observed difference across all models.

The resulting CL-GEMs contained 5,297 to 5,584 reactions associated with 2,193 to 2,328 genes and involving 4,209 to 4,432 metabolites (Table 1, Supplementary Dataset 3). In total 2,774 genes, 4,663 metabolites and 6,127 reactions were shared between all models from which 64.8% (n = 1798) of genes, 85.2% (n = 3975) of metabolites and 78.3% (n = 4798) of reactions were present in all of the CL-GEMs. We found 149 cell line-specific genes, 36 cell line-specific metabolites and 124 cell line-specific reactions were distributed between models, where models for hepatocellular carcinoma cell line (Hep-G2), osteosarcoma cell line (U-2 OS), urinary bladder transitional cell carcinoma cell line (RT-4) and colon adenocarcinoma cell line (CACO-2) contained nearly 70% of all the cell line-specific genes (Figure 2A). Pair wise comparison of the CL-GEMs revealed that each model has an average of 353 genes, 272 metabolites and 517 reactions being different from other cell lines, whereas the Hep-G2 and glioblastoma cell line (U-251 MG) showed highest degree of difference based on genes and reactions, respectively (Figure 2B, Supplementary Dataset 4). We also analyzed the transcript expression pattern of CL-GEMs and observed an average expression level of 34 to 58 FPKM for all genes incorporated into the metabolic models, approximately 4 fold higher than the expression level of cell line-specific genes (Supplementary Dataset 3), which shows that metabolism represents a core function in the cell lines.

We used CORE metabolite profiles of cancer cell lines^1^ to validate the functionality of four CL-GEMs (PC-3, A-549, MCF-7 and U-251) that were common in both studies. Constraining and optimizing the CL-GEMs based on CORE profiles, we observed that all models obtained a feasible solution with flux distribution consistent with published experimental data (Supplementary Dataset 5).

### Heterogeneity of CL-GEMs

**Figure 3:**
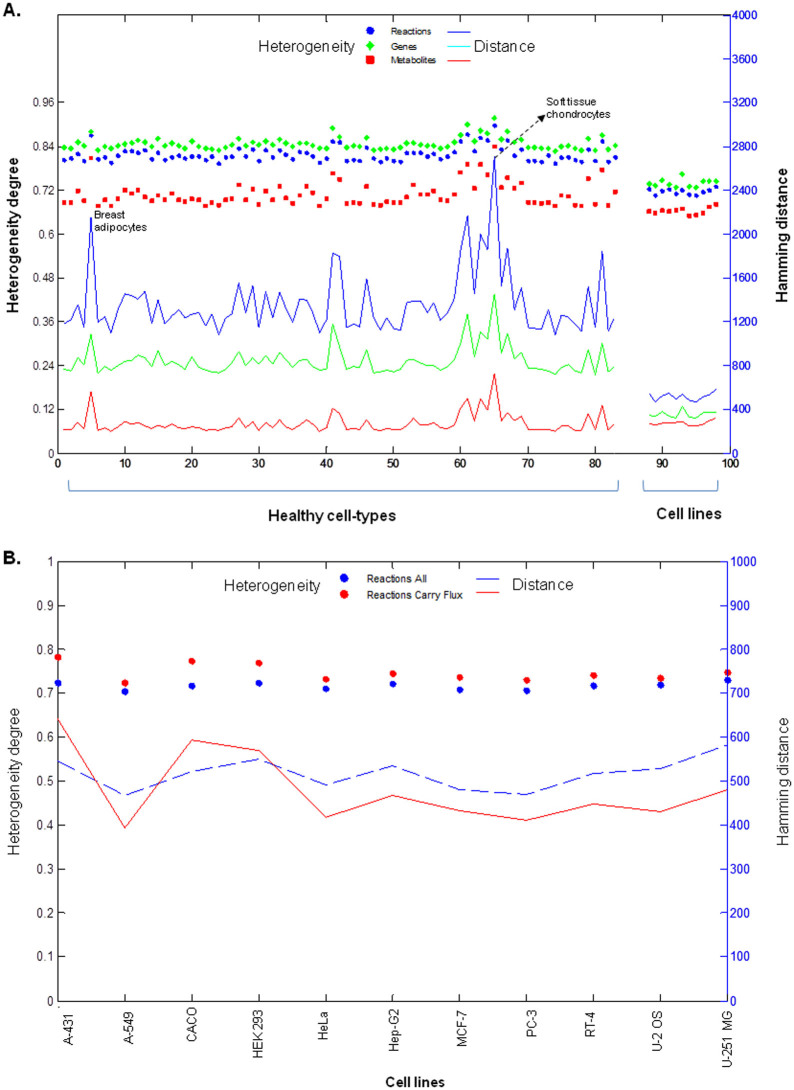
The heterogeneity of healthy cell-types and human cancer cell lines. (A) Heterogeneity degrees of GEMs for 83 healthy cell-types and cancer cell lines are projected on the left hand side axis. There is a relatively low, 0.07 degree, tendency towards uniformity of metabolic networks in cell lines comparing to healthy cell-types. Both GEMs for healthy cell-types and cancer cell lines show higher heterogeneity for genes and reactions compared to metabolites. However, the heterogeneity of models based on metabolites is stable, in contrast to reactions and genes. Hamming distances of GEMs for 83 healthy cell-types and CL-GEMs are projected on the right hand side axis. There is relatively high, ~50%, fall in Hamming distance for cell lines compared to healthy cell-types as result of tremendous dimensional variations, ~4 fold change, in metabolic models of healthy cell-types. In general, models show higher heterogeneity based on genes and larger distance based on reactions. (B) Heterogeneity of flux carrying as well as all reactions in CL-GEMs is shown on the left hand side axis. Hamming distances of CL-GEMs with flux carrying and all reactions are projected on the right hand side axis. Even though the content of the models decreased after removal of non-flux carrying reactions, the heterogeneity of the models was slightly increased.

Cancer cells reprogram their metabolic network to support cell proliferation by synthesizing all the cellular components and the required Gibbs free energy^16^. Despite the shared ability of transformed cells to induce growth stimulating adaptations towards uniformity of metabolism, significant marks of diversity and complexity in metabolic signatures of neoplasms has been recognized in recent years^29^,^30^. In order to understand the heterogeneity of metabolic networks in cancer, we used the reconstructed CL-GEMs and analyzed the transformability of models based on differences in their constituent parameters: genes, metabolites and reaction. We calculated the average Hamming distance and defined a heterogeneity scale, independent of model size and parameter type, for CL-GEMs and compared them with the GEMs for 83 healthy cell-types (see M&M). Throughout the comparison, healthy cell-types showed an average heterogeneity degree of 0.85 for genes, 0.70 for metabolites and 0.80 for reactions whereas cell lines showed an average heterogeneity degree of 0.78 for genes, 0.69 for metabolites and 0.74 for reactions (Figure 3A). Higher heterogeneity degree of genes compared to reactions indicates the preference of parameter effect to dimension effect, considering the fact that the number of reactions in each model was in average 2.4 fold more than the number of genes (Supplementary Dataset 3). A fall in heterogeneity degree, approximately 0.07 degree based on genes and reactions, comparing healthy cell-types to cell lines revealed a tendency towards the uniformity of metabolic networks in cell lines. However, a significant transformation was not observed and the models maintained their heterogeneity. Interestingly, the heterogeneity degree of CL-GEMs based on metabolites was stable, in contrast to genes and reactions, and this indicated the preserved capability of altered metabolic networks to transform the metabolites similar to healthy cell-types.

We also checked the number of the flux carrying reactions in each model (Table 1) and found that the models show higher heterogeneity based on the flux carrying reactions even though the number of the reactions in the models decreased by ~2 fold (Figure 3B and Supplementary Dataset 6). This analysis clearly demonstrated the difference in the metabolism of the cancer cell lines.

Next, using the transcriptome expression profile of genes included into the CL-GEMs, we investigated the global shifts between the cell lines. Kruskal-Wallis one-way analysis of variance^31^ was employed to compare the distribution of mRNA expression pattern between models and calculated P-value = 0.0029 rejected the hypothesis of similarity of distributions at significance level of α = 0.01. Furthermore, we performed pair wise comparisons of CL-GEMs using Fisher's LSD test^32^ with a confidence level of 0.05 (Supplementary Dataset 7). We found that Hep-G2 is the cell line with highest rate of significant difference compared to the other cell lines, and all models except Lung carcinoma cell line (A-549) and CACO-2 cell lines had a significant difference compared to the Hep-G2 model. The A-549 and CACO-2 models were also significantly different from U-251 MG and U-2 OS models.

Thus, comparing GEMs through constituent parameters (genes, metabolites and reactions) and also using mRNA expression pattern disclosed a considerable difference between metabolic models for the eleven cell lines. This indicated the necessity of developing fine-tuned CL-GEMs to capture the metabolic alterations that are specific to each cancer cell line.

### Heterogeneous expression of metabolic subsystems

**Figure 4:**
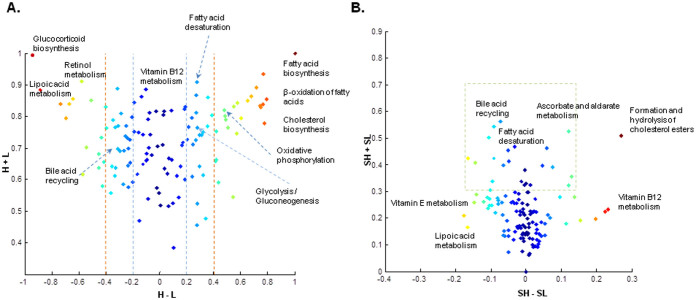
Metabolic subsystems in CL-GEMs. (A) Transcriptome expression pattern of metabolic subsystems in CL-GEMs are demonstrated. Metabolic subsystems are shown in H − L and H + L coordinates, where H is the fraction of models in which a subsystem has average high RNA expression level, and L is the fraction of models in which a subsystem has average low RNA expression level. Subsystems with dominantly high expression pattern clustered on the upper right corner of the plot, whereas subsystems with dominantly lower expression pattern occupy position in the upper left corner. Subsystems with heterogeneous behavior across cell lines are placed in the upper middle near zero on horizontal axis. Red dashed lines shows 40% of horizontal expansion and the blue dashed lines represent 20% of horizontal deviation. (B) Inter-cell line variation of heterogeneity in metabolic subsystems are demonstrated. Subsystems are shown in SH − SL and SH + SL coordinates, where for each pathway SH is the standard deviation of high RNA expression level across cell lines, and LH is the standard deviation of low RNA expression level. Subsystems with more variation in high RNA expression level occupy positions in the right-hand side of zero on horizontal axis, whereas subsystems with more variation in low RNA expression level placed in left-hand side of zero on horizontal axis. Subsystems with less expressional variation across cell lines are clustered in the lower part of the plot near zero on the horizontal axis. Heterogeneity mainly happens in the upper and upper right areas within the plot which are marked with dashed lines.

We analyzed the differences in mRNA expression pattern of the individual pathways across the eleven cell lines. We calculated the fraction of cell lines in which each metabolic subsystem had an average high transcript expression (H) and low transcript expression (L), and transferred the results into the (H − L) and (H + L) coordinates to observe the behavior of subsystems across cell lines (Figure 4A). We found highly, lowly and heterogeneously expressed subsystems in all eleven cell lines, and this allowed us to focus on subsystems that can be used for identifying generic or cell line-specific targets. As expected, biosynthesis pathways that are essential for biomass production and cell division displayed high expression levels in most of the cell lines, from which fatty acid biosynthesis, β-oxidation of fatty acids and cholesterol biosynthesis had the highest fraction of the H. Along with these pathways, ROS detoxification, folate metabolism and aminoacyl-tRNA biosynthesis displayed frequent high expression pattern in many of cell lines.

In contrast, lipoic acid metabolism and glucocorticoid biosynthesis revealed a relatively high fraction of L across all cell lines, which followed by retinol metabolism and androgen metabolism. Lipoic acid has been shown to reduce the proliferation/viability and to increase apoptosis of tumor cells^33^. Moreover, it has been reported that deficiency of lipoic acid synthetase results in defected mitochondrial energy metabolism and glycine cleavage mechanism as reflected by lactic acidosis and elevation of glycine^34^,^35^. Glucocorticoids have been used to treat wide range of cancers based on their substantial effect on progression of cell cycle and apoptosis through glucocorticoid receptor mediated mechanisms^36^,^37^,^38^. Retinoids are used for cancer treatment because of their ability to promote cell differentiation and also to change the gene expression pattern of tumor cells making them more vulnerable to other therapies^39^,^40^,^41^.

We next studied the inter-cell expressional variation of subsystems to explore the heterogeneous behavior of metabolic pathways across the different cell lines. Bile acid recycling, fatty acid desaturation, ascorbate and aldarate metabolism, formation and hydrolysis of cholesterol esters, and vitamin D metabolism showed higher heterogeneity among all subsystems in HMR2 (Figure 4B). In CACO-2, cervical epithelial adenocarcinoma cell line (HeLa) and Hep-G2, genes involved in bile acid recycling pathway mainly had low mRNA expression level, whereas in A-431 and RT-4, these genes had high expression levels. Almost all genes associated with reactions in fatty acid desaturation pathways were highly expressed in CACO-2 and Hep-G2 without showing a considerable high or low expression pattern for other cell lines. Formation and hydrolysis of cholesterol esters pathway was completely dominated by highly expressed genes for A-431 and A-549 different from other cell lines. Vitamin D metabolism revealed a different genes expression pattern for U-251 MG with low transcript level for all associated genes which followed by RT-4 and embryonal kidney cell line (HEK 293) with more than 70% of genes with low expression level. These analyses suggested that the activities of heterogeneous pathways are not influenced only by variability of environmental conditions, but also by the specific genetic repertoire of each individual cell line.

### Anti-growth Factors for Cell Lines

We used reconstructed CL-GEMs to predict the potential antigrowth factors through analyzing the robustness of metabolic networks against the induced perturbations. The metabolite-centric approach based on the concept of antimetabolites was employed to introduce the growth inhibiting perturbations. By testing the essentiality of metabolites present in the HMR2 on growth of cancer cell lines, we predicted 138 antimetabolites that can kill the growth on any of eleven studied cell lines and 106 of them were found to be effective in all cell lines. However, using these antimetabolites for the treatment of the cancer may have toxic effects to healthy cell-types in the human body. Therefore, we performed *in silico* toxicity test using the previously published GEMs for 83 different cell types in the human body. We checked if the use of the antimetabolites would potentially disrupt the metabolic tasks in energy production.

We finally predicted 85 potential antimetabolites that can inhibit growth of any cell lines studied here, while not being toxic to healthy cells (Supplementary Dataset 8). We found that 70.6% (n = 60) were effective in all cell lines, 11.8% (n = 10) were effective in two cell lines, 17.6% (n = 15) were effective in just one cell line, and interestingly not any antimetabolite was detected for 3 to 10 combinations of cell lines (Supplementary Dataset 9). Although ~70% of antimetabolites were predicted to be effective in all cell lines, the fact that 30% were cell-line specific indicated the importance of using cell line specific models rather than depending on one generic cancer model.

**Figure 5:**
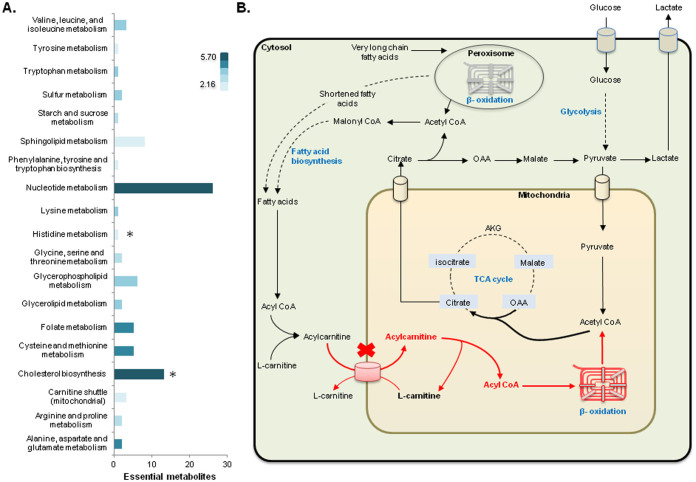
Prediction of antimetabolites through the use of CL-GEMs. (A) Distribution of 85 predicted antimetabolites and their corresponding metabolic subsystems in HMR2 are presented. Color gradient shows the average RNA expression level of related pathways in log2 scale. Stars indicate the two pathways with highest and lowest expression levels. (B) L-carnitine was predicted as an essential metabolite, and the use of its analogue was proposed for inhibiting the growth in all eleven cell lines. The predicted mechanism of action of an L-carnitine analogue is presented. L-carnitine antimetabolite may reduce β-oxidation and *de novo* fatty acids synthesis which is required for synthesis of the cell membrane and for cell proliferation. The abbreviations and the detailed explanations for the metabolites as well as the associated genes for each reaction are presented in HMR2.

Furthermore, we categorized 85 predicted antimetabolites based on their relevant subsystems in HMR2 and calculated the average mRNA expression level of the subsystems across cell lines (Figure 5A, Supplementary Dataset 10). It was observed that nucleotide metabolism, amino acids metabolism and cholesterol biosynthesis contain the highest fraction of antimetabolites, more than 68%. This was followed by sphingolipid, glycerophospholipid and folate metabolism. In general, pathways containing higher fraction of predicted antimetabolites showed high average mRNA expression levels in all cell lines and the exceptions were closely connected to one or more pathways with high average expression levels. For example, the carnitine shuttle had relatively low average expression level, but deficiency in this pathway results in deficiency of the β-oxidation pathway that has one of highest average RNA expression levels within all cell lines.

Following the prediction of L-carnitine as an antimetabolite and considering the connection of the L-carnitine shuttle to the mitochondrial β-oxidation, the analogue of L-carnitine were proposed as a potential antigrowth factor targeting the growth in all eleven cell lines (Figure 5B). L-carnitine is an essential metabolite that plays an important role in fatty acids metabolism and energy production. In humans, carnitine homoeostasis is preserved by dietary absorption and also through a multi-step *de novo* biosynthesis^42^. L-carnitine can be synthesized in liver and kidney using essential amino acids lysine and methionine as primary precursors to form trimethyllysine (TML)^43^,^44^. Notably, both lysine and methionine were also detected as antimetabolites through our analysis.

β-Oxidation was found to have high average mRNA expression levels in all eleven cell lines. This pathway consists of recurrent series of reactions that result in cyclical shortening of fatty acids and production of acetyl CoA, NADH and FADH. Generated NADH and FADH are used for ATP production through electron transport chain, while acetyl CoA enters the citric acid cycle (TCA)^44^,^45^,^46^. Carnitine shuttle acts as the carrier for the long chain fatty acid acyl groups and transports them from the cytosol to the mitochondria, where β-oxidation occurs. L-carnitine has an important role in transfer of the acetyl-CoA produced by peroxisomal β-oxidation to the mitochondria for oxidation. Other functions performed by L-carnitine include storing energy as acetylcarnitine, regulating the ratio of acyl-CoA to CoA and exerting the poorly metabolized acyl groups as carnitine esters to adjust their toxic effects^42^,^43^,^44^,^47^,^48^.

**Figure 6:**
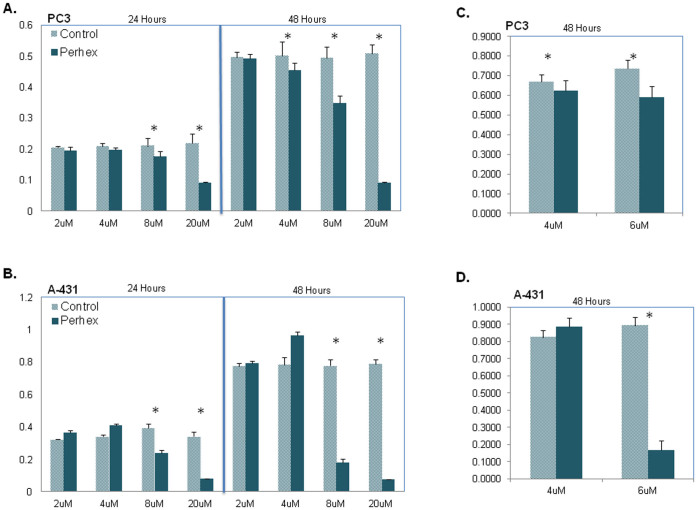
Inhibitory effect of Perhexiline on the proliferation of cell lines. Effect of Perhexiline on the proliferation of prostate carcinoma cell line, PC-3 and epidermoid carcinoma cell line, A-431 are shown. Perhexiline was used to mimic the L-carnitine analogue mechanism of effect on the proliferation of PC-3 and A-431 cell lines. (A) PC-3 and (B) A-431 cell lines were treated by 2, 4, 8 and 20 μM of Perhexiline and viability of cells determined after 24 and 48 hours. Perhexiline was dissolved in DMSO, and corresponding concentrations of DMSO in the medium were used as controls. Results of analyzing eight replicates of all concentrations and corresponding controls were represented by bar plots, mean ± standard deviation. Significantly difference between control and treated cell line: * (Student's t-test, p-value < 0.05). (C) PC-3 and (D) A-431 cell lines were treated by 4 and 6 μM of Perhexiline and viability of cells determined after 48 hours. Results of analyzing 16 replicates of two concentrations and corresponding controls were represented by bar plots, mean ± standard deviation. Significantly difference between control and treated cell line: * (Student's t-test, p-value < 0.05).

The use of L-carnitine analog inhibits the carnitine palmitoyltransferase (CPT) 1 and 2 enzymes that catalyze the L-carnitine and fatty acyl CoAs. We used perhexiline malate salt (perhexiline) that inhibits CPT1 and partly CPT2 to mimic the effect of the potential L-carnitine antimetabolite. To confirm our prediction about the use of L-carnitine analogue as potential antigrowth factor for all cell lines, we tested the effect of Perhexiline on the proliferation of prostate carcinoma cell line (PC-3) and A-431. Perhexiline was used to imitate the L-carnitine mechanism of effect since mitochondrial enzyme CPT1, as a member of carnitine acyltransferases family of enzymes, is responsible for translocation of conjugated L-carnitine and long chain FAs from cytosol to the mitochondria (Figure 5B). We treated two cell lines with four different concentrations of Perhexiline (2, 4, 8 and 24 μM) and investigated the viability of cells after 24 and 48 hours with eight replicates (Figure 6A–B). For lower concentration of Perhexiline (4 and 6 μM), we repeated our experiments with 16 replicates for 48 hours (Figure 6C–D). Significant reduction (t-test, p-value 0.05) in cell lines viability was observed with Perhexiline concentrations more than 2 μM for PC-3 and 4 μM for A-431.

**Figure 7:**
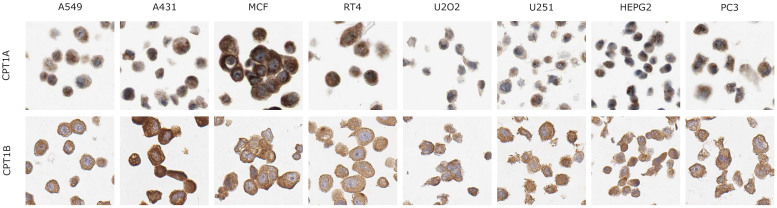
Protein staining of CPT1 and CPT2 in cell lines. Protein staining level of CPT1 and CPT2 are shown using the antibodies generated in Human Protein Atlas. Protein expression is seen in brown, counterstaining in blue.

We also analyzed protein expression level of CPT1 and CPT2 using the antibodies generated in the HPA project^49^ and observed that CPT1 and CPT2 had strong or moderate protein expression levels. Protein expression levels as detected by using immunoshistochemistry are exemplified in a subset of cell lines in Figure 7.

## Discussion

Recent advances in understanding cancer cell biology and continuous development of high throughput analytical methods have revealed that cancer is remarkably complex and heterogeneous. Increased understanding of this genomic heterogeneity between different tumors may open a new window towards the development and optimization of therapeutic methods for specific cancer types. GEMs provide a mechanistic description of relationships between genes and metabolic functions, and they can therefore be used for the interpretation of large experimental datasets. In this study we reconstructed CL-GEMs for eleven human cancer cell lines based on RNA expression data and used the generated models to understand the heterogeneity of metabolic networks in cancer cell lines. Our analysis revealed notable heterogeneity across CL-GEMs, where each model had an average of 353 different genes (heterogeneity degree of 0.78), 272 different metabolites (heterogeneity degree of 0.69) and 517 different reactions (heterogeneity degree of 0.74) in comparison to other models. Comparing CL-GEMs with GEMs for 83 healthy cell-types, we found a slight tendency towards the uniformity of metabolic networks in CL-GEMs and the heterogeneity of models preserved to a large extent. This finding indicates inadequacy of reconstructing a generic cancer model to capture metabolic alterations in transformed cells.

We analyzed the RNA expression pattern of individual pathways to identify the transcriptome based distribution of subsystems across CL-GEMs. We found that genes involved in fatty acid biosynthesis and cholesterol biosynthesis had highest average mRNA expression level in most of the cell lines, which shows the importance of biosynthesis pathways in transformed cells. On the other hand, lipoic acid metabolism and glucocorticoid biosynthesis revealed lowest average expression level among subsystems consistent with previously published studies indicating their positive correlation to reduced cell proliferation/viability and increased apoptosis of tumor cells^33^. Through in-depth analysis of expressional variation of subsystems across CL-GEMs, we found that bile acid recycling, fatty acid desaturation, ascorbate and aldarate metabolism, formation and hydrolysis of cholesterol esters, and vitamin D metabolism are the pathways with highest expressional heterogeneity among all subsystems in HMR2. Subsystems with high expressional variation may be potential targets for further studies to find cell line specific targets.

Metabolite essentiality, defined as metabolites necessary for cell growth and survivor, has previously been used^50^ to identify potential drug targets for an opportunistic pathogen using GEMs. We used this concept to explore essential metabolites in CL-GEMs by perturbing the metabolic networks and found metabolites contributing to growth inhibiting perturbations. The structural analogues of 85 predicted essential metabolites, which passed the *in silico* toxicity test based on previously generated GEMs for 83 healthy human cell types, proposed as potential antimetabolites. Analogues of L-carnitine were proposed as potential antimetabolites, due to their key role in L-carnitine shuttle pathway, and the contribution of L-carnitine shuttle to the mitochondrial β-oxidation and consequently to the fatty acid synthesis. Two cell lines with different phenotypic origins, PC-3 and A-431 were selected for *in vitro* testing of our prediction using Perhexiline to mimic the effect of L-carnitine analogue. Both cell lines revealed significant reduction in cells viability in presence of Perhexiline, validating the relevancy of predicted target. The effect of Perhexiline on viability of Hep-G2 has previously been verified by ref. 15, and is consistent with our findings.

Plasticity is one of the key factors to maintain fitness of transformed cells and β-oxidation of fatty acids may prepare some of this plasticity by supplying metabolic intermediates for cell proliferation, suppressing pro-apoptotic subsystems, eradicating potentially cytotoxic lipids, and empowering the production of NADPH and ATP when required^51^,^52^,^53^,^54^,^55^. However, β-oxidation of fatty acids cannot be discerned as an independent metabolic pathway in cancer metabolism and there is a challenge to unify the essential role of β-oxidation pathway in tumor cells with the fact that active fatty acid synthesis is necessary for cancer cells division and proliferation.

Despite promising performance of GEMs in identification of biomarkers and drug targets for cancer, there are cautions and limitations regarding this approach. Unlike bacteria, reconstructing and testing the functionality of GEMs is more complex when it comes to cells and tissues. Constructing new generation of unified GEMs that integrate metabolism with other cellular processes, especially signaling and regulation, remains as a nontrivial challenge. Different assumptions to estimate the connection between transcription, translation and flux rate challenges the accuracy and compatibility of models generated by incorporating omics data into GEMs.

In conclusion, we used CL-GEMs to investigate the differences in the metabolism of cell lines and explore potential antigrowth factors, which may impede the growth of cancer cells, and experimentally verified the inhibitory effect of the L-carnitine analogue, one of the predicted antimetabolite. The study of cancer metabolism using GEMs has revealed new therapeutic opportunities, as well as more profound understanding of metabolic reprogramming of cancer cells. Over the past decades we learned that targeting single pathways or enzymes is not enough to cure cancer and agents proposed here as antigrowth factors may be combined with other therapeutic agents and methods to get desired results. The approach presented here may also be extended to study the combinatory effect of potential and standard therapeutic agents to find more effective cancer specific therapies, and may present new possibilities towards personalized medicine.

## Methods

### GEM reconstruction

The tINIT algorithm^15^ implemented in the RAVEN toolbox^56^ was used for the reconstruction of eleven CL-GEMs. 56 Metabolic functions known to occur in all cell lines as well as growth were used during the reconstruction process of the GEMs (Supplementary Dataset 2). During the reconstruction of the model, we used the content of Ham's media as minimal media required for cell growth. The content of the Ham's media is provided in Supplementary dataset 2.

### Heterogeneity of CL-GEMs

A new measure of heterogeneity was used in our study to capture the divergence between metabolic networks based on their constituent parameters: genes, reactions and metabolites. For each model, heterogeneity degree formulated as:
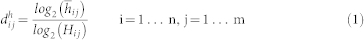
Where *d^h^_ij_* is the heterogeneity degree of model i based on parameter j, 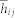 is the average Hamming distance of model i to all other models based on parameter j, 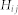 is maximum Hamming distance of model i based on parameter j in comparison to integrated vector of parameter j (*Iv_j_*). In this formulation, n is the number of models (83 for healthy cell-types and 11 for cancer cell lines), m is the number of the constituent parameters (here parameter are genes, reaction and metabolites, m = 3), and *Iv_j_* is a unique union of corresponding parameter across all models. For example, *I.v*_1_ represent a union vector of genes present at least in one of the models.

To compare the CL-GEMs based on RNA expression levels, we built a vector of expected transcriptome expression values of genes linked to metabolic subsystems for each model, and merged these vectors into n × m unified ratio matrix. Here, n is number of subsystems and m is the number of the models. We used this matrix to perform multi-comparison analysis employing Kruskal-Wallis non-parametric test with significance level of 0.01. Finally, we performed pair wise analysis of models using post hoc Fisher's LSD test with confidence level of 0.05.

### Heterogeneous expression of metabolic pathways

To identify the heterogeneous expression of metabolic subsystems across the cell lines, we calculated the ratios of high and low RNA expression level for genes associated to reactions inside the CL-GEMs in logarithmic scale. For each pathway, we calculated the average ratio of high expression level and low expression level and integrated the results into two different n × m ratio matrices, high matrix (HM) and low matrix (LM), respectively. Here, n is number of pathways and m is the number of models. We used a subsystem-specific metrics, HM-LM and HM + LM, to compare the expression pattern of pathways across the cell lines.

### In *silico* toxicity test

Inhibition of essential metabolites in cancer cell lines can also disrupt the metabolism of healthy cells, similar to chemotherapeutic drugs. To avoid this side effect, we used reconstructed GEMs for 83 healthy cell types and investigated the response of these metabolic models against inhibition of essential metabolites. We excluded the essential metabolites that disrupted energy metabolism and redox balance in healthy cells. Our approach was based on the central role of energy producing tasks for all cell types, and also high likelihood of neoplastic shifts in energy metabolism of transformed cells.

### Effect of Perhexiline on PC-3 and A-431 cell lines

A proliferation assay was performed using the colorimetric CellTiter 96® AQ_ueous_ One Solution Cell Proliferation Assay (MTS) (Promega) on PC-3 and A-431 according to instructions from the manufacturer (DSMZ, Braunscweig, Germany). In brief, the two cell lines were treated with 2, 4, 8 and 20 μM of Perhexiline Malate salt (Sigma-Aldrich, St. Louis, USA) for 24 and 48 hours respectively. Perhexiline was dissolved in DMSO, and corresponding concentrations of DMSO in medium were used as controls. In addition, controls consisting of cells growing in only cell culture medium were included. For all concentrations and corresponding DMSO-controls eight replicates were analysed, and for the controls consisting of cells in only medium 16 replicates were analysed. In the follow up experiment the two cell lines were treated with 4 and 6 μM respectively of Perhexiline Malate salt (Sigma-Aldrich, St. Louis, USA) for 48 hours. This time 16 replicates were used for all conditions and controls.

## Author Contributions

P.G. reconstructed the models and P.G. and A.M. performed the analysis of the data. A.A., C.K. and M.U. performed the *in-vitro* experiments. S.S. validated the functionality of the models. J.N. and M.U. conceived the project. P.G., A.M. and J.N. wrote the paper and all authors were involved in editing the paper.

## Supplementary Material

Supplementary Information Supplementary Material

Supplementary Information Supplementary Dataset 1-10

## Figures and Tables

**Table 1:** The reactions, metabolites and genes included in the CL-GEMs

CL-GEMs	GENES	Reactions	Reactions Carry Flux	Metabolites
A-431 *Epidermoid carcinoma cell line*	2193	5297	2914	4245
A-549 *Lung carcinoma cell line*	2318	5574	3335	4382
CACO-2 *Colon adenocarcinoma cell line*	2271	5472	2955	4326
HEK 293 *Embryonal kidney cell line*	2287	5274	2856	4220
HeLa *Cervical epithelial adenocarcinoma cell line*	2267	5570	3340	4397
Hep-G2 *Hepatocellular carcinoma cell line*	2273	5646	3431	4432
MCF-7 *Metastatic breast adenocarcinoma cell line*	2243	5402	3153	4318
PC-3 *Prostate adenocarcinoma cell line*	2297	5431	3328	4263
RT-4 *Urinary bladder transitional cell carcinoma cell line*	2251	5584	3326	4407
U-2 OS *Osteosarcoma cell line*	2328	5452	3221	4333
U-251 *MG Glioblastoma cell line*	2194	5320	3292	4209

## References

[b1] Jain M. *et al.* Metabolite Profiling Identifies a Key Role for Glycine in Rapid Cancer Cell Proliferation. Science 336, 1040–1044 (2012). 22628656 10.1126/science.1218595 PMC3526189

[b2] Moghaddas Gholami A. *et al.* Global proteome analysis of the NCI-60 cell line panel. Cell Rep 4, 609–620 (2013). 23933261 10.1016/j.celrep.2013.07.018

[b3] Geiger T., Wehner A., Schaab C., Cox J. & Mann M. Comparative proteomic analysis of eleven common cell lines reveals ubiquitous but varying expression of most proteins. Mol Cell Proteomics 11, M111 014050 (2012). 10.1074/mcp.M111.014050 PMC3316730 22278370

[b4] Hyduke D. R., Lewis N. E. & Palsson B. O. Analysis of omics data with genome-scale models of metabolism. Molecular bioSystems 9, 167–174 (2013). 23247105 10.1039/c2mb25453k PMC3594511

[b5] Mardinoglu A. & Nielsen J. Systems medicine and metabolic modelling. Journal of internal medicine 271, 142–154 (2012). 22142312 10.1111/j.1365-2796.2011.02493.x

[b6] Mardinoglu A., Gatto F. & Nielsen J. Genome-scale modeling of human metabolism - a systems biology approach. Biotechnology journal 8, 985–996 (2013). 23613448 10.1002/biot.201200275

[b7] Shoaie S. & Nielsen J. Elucidating the interactions between the human gut microbiota and its host through metabolic modeling. Front Genetics 5, 86 (2014). 10.3389/fgene.2014.00086 PMC4000997 24795748

[b8] Duarte N. C. *et al.* Global reconstruction of the human metabolic network based on genomic and bibliomic data. Proc Natl Acad Sci U S A 104, 1777–1782 (2007). 17267599 10.1073/pnas.0610772104 PMC1794290

[b9] Thiele I. *et al.* A community-driven global reconstruction of human metabolism. Nat Biotechnol 31, 419–425 (2013). 23455439 10.1038/nbt.2488 PMC3856361

[b10] Mardinoglu A. *et al.* Integration of clinical data with a genome-scale metabolic model of the human adipocyte. Mol Syst Biol 9, 649 (2013). 23511207 10.1038/msb.2013.5 PMC3619940

[b11] Mardinoglu A. *et al.* Genome-scale metabolic modelling of hepatocytes reveals serine deficiency in patients with non-alcoholic fatty liver disease. Nature communications 5, 3083 (2014). 10.1038/ncomms4083 24419221

[b12] Agren R. *et al.* Reconstruction of Genome-Scale Active Metabolic Networks for 69 Human Cell Types and 16 Cancer Types Using INIT. Plos Comput Biol 8, e1002518 (2012). 22615553 10.1371/journal.pcbi.1002518 PMC3355067

[b13] Frezza C. *et al.* Haem oxygenase is synthetically lethal with the tumour suppressor fumarate hydratase. Nature 477, 225–228 (2011). 21849978 10.1038/nature10363

[b14] Gatto F., Nookaew I. & Nielsen J. Chromosome 3p loss of heterozygosity is associated with a unique metabolic network in clear cell renal carcinoma. P Natl Acad Sci USA 111, E866–E875 (2014). 10.1073/pnas.1319196111 PMC3948310 24550497

[b15] Agren R. *et al.* Identification of anticancer drugs for hepatocellular carcinoma through personalized genome-scale metabolic modeling. Mol Syst Biol 10, 721 (2014). 24646661 10.1002/msb.145122 PMC4017677

[b16] Hanahan D. & Weinberg R. A. Hallmarks of cancer: the next generation. Cell 144, 646–674 (2011). 21376230 10.1016/j.cell.2011.02.013

[b17] Ward P. S. & Thompson C. B. Metabolic Reprogramming: A Cancer Hallmark Even Warburg Did Not Anticipate. Cancer Cell 21, 297–308 (2012). 22439925 10.1016/j.ccr.2012.02.014 PMC3311998

[b18] Lazar M. A. & Birnbaum M. J. Physiology. De-meaning of metabolism. Science 336, 1651–1652 (2012). 22745413 10.1126/science.1221834

[b19] Kaye S. B. New antimetabolites in cancer chemotherapy and their clinical impact. British journal of cancer 78 Suppl 3, 1–7 (1998). 9717984 10.1038/bjc.1998.747 PMC2062805

[b20] Hebar A., Valent P. & Selzer E. The impact of molecular targets in cancer drug development: major hurdles and future strategies. Expert review of clinical pharmacology 6, 23–34 (2013). 23272790 10.1586/ecp.12.71

[b21] Garg D. *et al.* Novel approaches for targeting thymidylate synthase to overcome the resistance and toxicity of anticancer drugs. Journal of medicinal chemistry 53, 6539–6549 (2010). 20527892 10.1021/jm901869w

[b22] Fagerberg L. *et al.* Contribution of antibody-based protein profiling to the human Chromosome-centric Proteome Project (C-HPP). Journal of proteome research 12, 2439–2448 (2013). 23276153 10.1021/pr300924j

[b23] Fagerberg L. *et al.* Analysis of the Human Tissue-specific Expression by Genome-wide Integration of Transcriptomics and Antibody-based Proteomics. Mol Cell Proteomics 13, 397–406 (2014). 24309898 10.1074/mcp.M113.035600 PMC3916642

[b24] Kampf C. *et al.* Defining the human gallbladder proteome by transcriptomics and affinity proteomics. Proteomics 14, 2498–2507 (2014). 25175928 10.1002/pmic.201400201

[b25] Mardinoglu A. *et al.* Defining the human adipose tissue proteome to reveal metabolic alterations in obesity. J Proteome Res 13, 5106–5119 (2014). 25219818 10.1021/pr500586e

[b26] Kampf C. *et al.* The human liver-specific proteome defined by transcriptomics and antibody-based profiling. Faseb J 28, 2901–2914 (2014). 24648543 10.1096/fj.14-250555

[b27] Lindskog C. *et al.* The lung-specific proteome defined by integration of transcriptomics and antibody-based profiling. Faseb J 28, 5184–5196 (2014). 25169055 10.1096/fj.14-254862

[b28] Folger O. *et al.* Predicting selective drug targets in cancer through metabolic networks. Mol Syst Biol 7, 501 (2011). 21694718 10.1038/msb.2011.35 PMC3159974

[b29] Cantor J. R. & Sabatini D. M. Cancer cell metabolism: one hallmark, many faces. Cancer discovery 2, 881–898 (2012). 23009760 10.1158/2159-8290.CD-12-0345 PMC3491070

[b30] Hu J. *et al.* Heterogeneity of tumor-induced gene expression changes in the human metabolic network. Nature biotechnology 31, 522–529 (2013). 10.1038/nbt.2530 PMC3681899 23604282

[b31] Kruskal W. H. & Wallis W. A. Use of Ranks in One-Criterion Variance Analysis. J Am Stat Assoc 47, 583–621 (1952).

[b32] Hayter A. J. The Maximum Familywise Error Rate of Fishers Least Significant Difference Test. J Am Stat Assoc 81, 1000–1004 (1986).

[b33] Feuerecker B. *et al.* Lipoic acid inhibits cell proliferation of tumor cells in vitro and in vivo. Cancer biology & therapy 13, 1425–1435 (2012). 22954700 10.4161/cbt.22003 PMC3542233

[b34] Goraca A. *et al.* Lipoic acid - biological activity and therapeutic potential. Pharmacological reports: PR 63, 849–858 (2011). 22001972 10.1016/s1734-1140(11)70600-4

[b35] Mayr J. A. *et al.* Lipoic acid synthetase deficiency causes neonatal-onset epilepsy, defective mitochondrial energy metabolism, and glycine elevation. American journal of human genetics 89, 792–797 (2011). 22152680 10.1016/j.ajhg.2011.11.011 PMC3234378

[b36] Schlossmacher G., Stevens A. & White A. Glucocorticoid receptor-mediated apoptosis: mechanisms of resistance in cancer cells. The Journal of endocrinology 211, 17–25 (2011). 21602312 10.1530/JOE-11-0135

[b37] Sionov R. V., Spokoini R., Kfir-Erenfeld S., Cohen O. & Yefenof E. Mechanisms regulating the susceptibility of hematopoietic malignancies to glucocorticoid-induced apoptosis. Advances in cancer research 101, 127–248 (2008). 19055945 10.1016/S0065-230X(08)00406-5

[b38] Spokoini R., Kfir-Erenfeld S., Yefenof E. & Sionov R. V. Glycogen synthase kinase-3 plays a central role in mediating glucocorticoid-induced apoptosis. Molecular endocrinology 24, 1136–1150 (2010). 20371704 10.1210/me.2009-0466 PMC5417474

[b39] Blomhoff R. & Blomhoff H. K. Overview of retinoid metabolism and function. Journal of neurobiology 66, 606–630 (2006). 16688755 10.1002/neu.20242

[b40] Gudas L. J. & Wagner J. A. Retinoids regulate stem cell differentiation. Journal of cellular physiology 226, 322–330 (2011). 20836077 10.1002/jcp.22417 PMC3315372

[b41] Tang X. H. & Gudas L. J. Retinoids, retinoic acid receptors, and cancer. Annual review of pathology 6, 345–364 (2011). 10.1146/annurev-pathol-011110-130303 21073338

[b42] Goa K. L., Brogden R. N. l-Carnitine. A preliminary review of its pharmacokinetics, and its therapeutic use in ischaemic cardiac disease and primary and secondary carnitine deficiencies in relationship to its role in fatty acid metabolism. Drugs 34, 1–24 (1987). 3308409 10.2165/00003495-198734010-00001

[b43] Pekala J. *et al.* L-Carnitine - Metabolic Functions and Meaning in Humans Life. Curr Drug Metab 12, 667–678 (2011). 21561431 10.2174/138920011796504536

[b44] Vaz F. M. & Wanders R. J. Carnitine biosynthesis in mammals. The Biochemical journal 361, 417–429 (2002). 11802770 10.1042/0264-6021:3610417 PMC1222323

[b45] Bartlett K. & Eaton S. Mitochondrial beta-oxidation. Eur J Biochem 271, 462–469 (2004). 14728673 10.1046/j.1432-1033.2003.03947.x

[b46] Jakobs B. S., Wanders R. J. A. Fatty-Acid Beta-Oxidation in Peroxisomes and Mitochondria - the First, Unequivocal Evidence for the Involvement of Carnitine in Shuttling Propionyl-Coa from Peroxisomes to Mitochondria. Biochem Bioph Res Co 213, 1035–1041 (1995). 10.1006/bbrc.1995.2232 7654220

[b47] Duran M., Loof N. E., Ketting D. & Dorland L. Secondary Carnitine Deficiency. J Clin Chem Clin Bio 28, 359–363 (1990). 2199597

[b48] McGarry J. D. & Brown N. F. The mitochondrial carnitine palmitoyltransferase system - From concept to molecular analysis. Eur J Biochem 244, 1–14 (1997). 9063439 10.1111/j.1432-1033.1997.00001.x

[b49] Uhlen M. *et al.* Towards a knowledge-based Human Protein Atlas. Nat Biotechnol 28, 1248–1250 (2010). 21139605 10.1038/nbt1210-1248

[b50] Kim H. U. *et al.* Integrative genome-scale metabolic analysis of Vibrio vulnificus for drug targeting and discovery. Mol Syst Biol 7, 460 (2011). 21245845 10.1038/msb.2010.115 PMC3049409

[b51] Carracedo A., Cantley L. C. & Pandolfi P. P. Cancer metabolism: fatty acid oxidation in the limelight. Nature reviews Cancer 13, 227–232 (2013). 10.1038/nrc3483 PMC3766957 23446547

[b52] Zaugg K. *et al.* Carnitine palmitoyltransferase 1C promotes cell survival and tumor growth under conditions of metabolic stress. Genes & development 25, 1041–1051 (2011). 21576264 10.1101/gad.1987211 PMC3093120

[b53] Samudio I. *et al.* Pharmacologic inhibition of fatty acid oxidation sensitizes human leukemia cells to apoptosis induction. The Journal of clinical investigation 120, 142–156 (2010). 20038799 10.1172/JCI38942 PMC2799198

[b54] Paumen M. B. *et al.* Direct interaction of the mitochondrial membrane protein carnitine palmitoyltransferase I with Bcl-2. Biochem Biophys Res Commun 231, 523–525 (1997). 9070836 10.1006/bbrc.1997.6089

[b55] Santos C. R. & Schulze A. Lipid metabolism in cancer. The FEBS journal 279, 2610–2623 (2012). 22621751 10.1111/j.1742-4658.2012.08644.x

[b56] Agren R. *et al.* The RAVEN toolbox and its use for generating a genome-scale metabolic model for Penicillium chrysogenum. PLoS computational biology 9, e1002980 (2013). 23555215 10.1371/journal.pcbi.1002980 PMC3605104

