# Supplementary material for: Identifying anti-growth factors for human cancer cell lines through genome-scale metabolic modeling
Source: Sci Rep. 2015 Feb 2;5:8183. doi: 10.1038/srep08183 (PMC4313100; doi:10.1038/srep08183)
Supplement: Supplementary Information — Supplementary Material [file srep08183-s1.doc]

**Identifying anti-growth factors for human cancer cell lines through genome-scale metabolic modeling**

**Running title: Anti-growth factors for human cancer cell lines**

Pouyan Ghaffari^1^, Adil Mardinoglu^1^, Anna Asplund^2^, Saeed Shoaie^1^, Caroline Kampf^2^, Mathias Uhlen^3,4^, Jens Nielsen^1,3^,*

^1^ Department of Chemical and Biological Engineering, Chalmers University of Technology, SE-412 96, Gothenburg, Sweden

^2^ Department of Immunology, Genetics and Pathology, Science for Life Laboratory, Uppsala University, SE-751 85, Uppsala, Sweden

^3^ Science for Life Laboratory, KTH - Royal Institute of Technology, SE-171 21, Stockholm, Sweden

^4^ Department of Proteomics, KTH - Royal Institute of Technology, SE-106 91, Stockholm, Sweden

*Corresponding author

E-mail: [nielsenj@chalmers.se](mailto:nielsenj@chalmers.se)

Tel: +46 31 772 3804

Fax: +46 31 772 3801

## SUPPLEMENTARY DATASET LEGENDS

**Dataset 1** Transcript expression level of protein coding genes distributed across eleven human cancer cell lines.

**Dataset 2** 56 metabolic tasks which must occur in all cell types and used to reconstruct cell line-specific genome scale metabolic models (CL-GEMs). These tasks categorized in five different groups: energy and redox (ER), biosynthesis of products (BS), substrate utilization (SU), internal conversions (IC) and biomass growth (GR).

**Dataset 3** Genes, reactions and metabolites in CL-GEM.

**Dataset 4** Pairwise comparison of CL-GEMs based on constituent parameters: genes, reactions and metabolites.

**Dataset 5** Flux distribution of CL-GEMs constrained and optimized based on consumption and release (CORE) profiles of cancer cell lines.

**Dataset 6** Heterogeneity analysis and the pairwise comparisons of the flux carrying reactions in CL-GEMs.

**Dataset 7** Pairwise comparisons of CL-GEMs using Fisher’s LSD test with confidence level of 0.05.

**Dataset 8** 85 predicted essential metabolites that, upon blockage, can inhibit growth of any cell lines studied here. For each essential metabolite corresponding HMR2 subsystem was also included.

**Dataset 9** The presence/absence of the predicted essential metabolites in eleven cell lines.

**Dataset 10** 85 predicted essential metabolites categorized based on their relevant subsystems in HMR2 and calculated the average mRNA expression level of the subsystems across cell lines.
